# MicroRNA Expression Profiling in CCl_4_-Induced Liver Fibrosis of *Mus musculus*

**DOI:** 10.3390/ijms17060961

**Published:** 2016-06-17

**Authors:** Jeongeun Hyun, Jungwook Park, Sihyung Wang, Jieun Kim, Hyun-Hee Lee, Young-Su Seo, Youngmi Jung

**Affiliations:** 1 Department of Biological Sciences, Pusan National University, Pusan 46241, Korea; j.hyun@pusan.ac.kr (J.H.); s.wang@ pusan.ac.kr (S.W.); jieun@pusan.ac.kr (J.K.); 2 Department of Microbiology, Pusan National University, Pusan 46241, Korea; jjuwoogi@pusan.ac.kr (J.P.); ehyuna92@pusan.ac.kr (H.-H.L.)

**Keywords:** microRNA, microarray, liver fibrosis, mouse, gene ontology

## Abstract

Liver fibrosis is a major pathological feature of chronic liver diseases, including liver cancer. MicroRNAs (miRNAs), small noncoding RNAs, regulate gene expression posttranscriptionally and play important roles in various kinds of diseases; however, miRNA-associated hepatic fibrogenesis and its acting mechanisms are poorly investigated. Therefore, we performed an miRNA microarray in the fibrotic livers of *Mus musculus* treated with carbon-tetrachloride (CCl_4_) and analyzed the biological functions engaged by the target genes of differentially-expressed miRNAs through gene ontology (GO) and in-depth pathway enrichment analysis. Herein, we found that four miRNAs were upregulated and four miRNAs were downregulated more than two-fold in CCl_4_-treated livers compared to a control liver. Eight miRNAs were predicted to target a total of 4079 genes. GO analysis revealed that those target genes were located in various cellular compartments, including cytoplasm, nucleolus and cell surface, and they were involved in protein-protein or protein-DNA bindings, which influence the signal transductions and gene transcription. Furthermore, pathway enrichment analysis demonstrated that the 72 subspecialized signaling pathways were associated with CCl_4_-induced liver fibrosis and were mostly classified into metabolic function-related pathways. These results suggest that CCl_4_ induces liver fibrosis by disrupting the metabolic pathways. In conclusion, we presented several miRNAs and their biological processes that might be important in the progression of liver fibrosis; these findings help increase the understanding of liver fibrogenesis and provide novel ideas for further studies of the role of miRNAs in liver fibrosis.

## 1. Introduction

Liver fibrosis, a typical feature of most chronic liver diseases, is characterized by excessive accumulation of extracellular matrix proteins, such as fibrillar collagen, as a result of the wound-healing response to persistent hepatic injury [1]. It accelerates the progression of liver disease by the destruction of the normal hepatic parenchyma, eventually leading to liver cirrhosis, liver failure or even liver cancer. Thus, reversing liver fibrosis is a critical point of therapy for patients with various chronic liver diseases with fibrosis, such as hepatitis B/C virus (HBV/HCV)-mediated cirrhosis, primary biliary cirrhosis and non-alcoholic steatohepatitis (NASH). Although several cytokines and signaling pathways involved in liver fibrosis have been found [1], it remains unclear how noncoding RNAs regulate the underlying mechanisms of hepatic fibrogenesis.

MicroRNAs (miRNAs) include approximately 22 nucleotides of endogenous noncoding RNAs conserved across species [2]. Newly-transcribed miRNAs have a relatively long stem-loop structure, and they are sequentially processed to become mature miRNAs by ribonuclease III enzymes Drosha and Dicer, found in the nucleus and the cytoplasm, respectively [2]. miRNAs interact with their target genes through base-pairing between their ~6–8 nucleotides in the 5′-end, called the seed sequence, and through the complementary sequences within the mRNA of the target genes, inducing degradation or translational inhibition of the mRNAs [3]. It has been reported that miRNAs are important regulators in various biological processes, such as cell proliferation, differentiation, immune response and metabolism [4,5,6], and their aberrant expression is closely associated with many diseases, including liver diseases [7,8]. Thus, miRNAs have come to the forefront as novel biomarkers and therapeutic agents for liver diseases.

Growing evidence suggests that dysregulated expression of miRNAs is closely associated with fibrosis appearing in various organs, including the liver [9,10]. Among those miRNAs, miR-21 and miR-29a/b are reported to be commonly related to fibrosis in multiple organs, including the heart, lung, skin, kidney and liver [9,10]. In the fibrotic liver, miR-21 is upregulated and promotes the transdifferentiation of quiescent hepatic stellate cells (HSCs) into myofibroblastic HSCs, a major cell type producing collagen in the liver [11]. miR-29a/b, which directly inhibits the synthesis of collagen, is downregulated during the activation of HSCs, promoting liver fibrosis [12]. In addition, miR-19b acts as an anti-fibrotic factor by regulating transforming growth factor (TGF)-β signaling in HSCs [13]. In the heart, miR-24 attenuates cardiac fibrosis and inhibits the differentiation of cardiac fibroblasts by targeting furin, a protease for TGF-β maturation [14]. miR-192 and miR-200a/b have antifibrotic roles in tubular epithelial cells, but they are profibrotic to the mesangial cells of the glomerulus in the kidney [10]. However, the studies reveal only part of the complicated mechanism of certain miRNA-mediated fibrogenesis. However, liver fibrosis is known with respect to the highly complicated physiological and molecular events. Thus, it is necessary to investigate the whole regulatory network composed of miRNA and its target genes to establish a better strategy for treating liver fibrosis.

There are currently 2603, 1920 and 807 known mature miRNAs in humans, mice and rats, respectively (miRBase, Release 21). These miRNAs were predicted to possibly bind with 19,475 (28,353 transcripts) genes in human (TargetScan, Release 7) and 18,615 (23,795 transcripts) genes in mice (TargetScan, Release 6). A recent study shows that more than one-third of human genes are regulated by miRNAs [6]. However, among these huge amounts of potential factors, it is difficult to investigate which miRNAs and which targets of a specific miRNA are important in the pathogenesis of disease. Therefore, microarray and functional analysis for their target genes are good methods to identify important miRNAs that play the essential roles in the pathological processes by regulating the expression of target genes.

In the present study, we analyzed the expressional level of genome-wide miRNAs in livers of carbon-tetrachloride (CCl_4_)- or corn oil-treated mice using the miRNA array assay. We found that a total of eight miRNAs were differentially expressed in fibrotic livers of CCl_4_-treated mice compared to the control livers of corn oil-treated mice (>2-fold, *p* < 0.05). A bioinformatics analysis showed that these miRNAs targeted 4079 potential genes. To identify the mechanisms regulated by these miRNAs during liver fibrosis, we conducted gene ontology (GO) analysis and in-depth Kyoto Encyclopedia of Genes and Genomes (KEGG) pathway enrichment analysis of the putative target genes. Seventy-two enriched signaling pathways were found to be associated with liver fibrosis (*p* < 0.05), and a total of 843 genes among 4079 potential target genes were shown to be involved in these enriched signaling pathways (*p* < 0.05). These enriched pathways were largely classified into metabolic-related pathways. These results demonstrate that differentially-expressed miRNAs in fibrotic livers target specific genes, being involved in metabolic-related pathways, suggesting that understanding the potential biological functions of these miRNAs in these pathways will help to develop novel therapeutics in treating patients with liver disease.

## 2. Results

### 2.1. Carbon-Tetrachloride-Induced Liver Fibrosis in Mice

**Figure 1:**
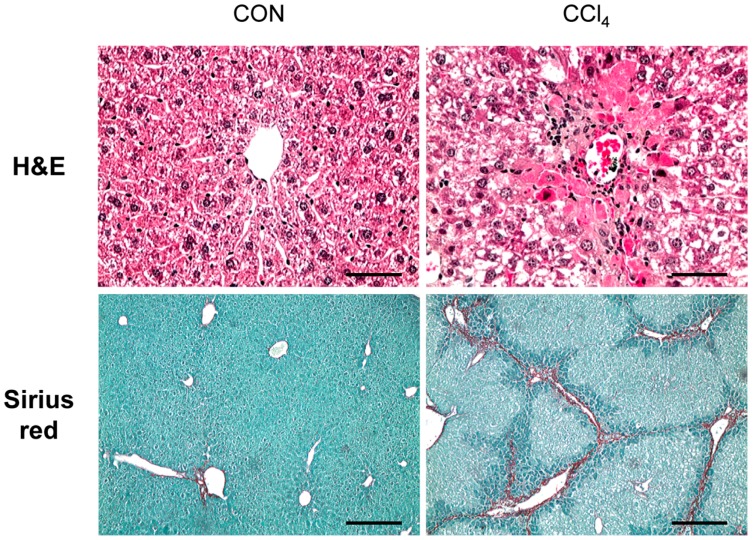
Changes of liver histomorphology and increased fibrosis in carbon-tetrachloride (CCl_4_)-treated livers. Hematoxylin-eosin (H&E) staining shows hepatocyte necrosis and immune-cell infiltration of the liver from representative CCl_4_-treated mice compared to corn oil-treated mice (CON) (scale bar = 50 µm). Sirius red staining in liver sections from representative CON and CCl_4_-treated mice shows the excessive collagen deposition in CCl_4_-treated livers (scale bar = 100 µm).

CCl_4_ is a well-known chemical that induces liver fibrosis through a hepatotoxic injury-mediated response in experimental animals [15,16]. To generate a chronically-damaged liver with fibrosis, we exposed mice to CCl_4_ for 10 weeks by intraperitoneal injection. Histomorphological changes after CCl_4_ treatment were examined by hematoxylin-eosin (H&E) stain (Figure 1). H&E staining showed that dead or ballooned hepatocytes were accumulated around the central vein, and immune cells infiltrated into the interspaces of these damaged hepatocytes in the liver sections of the CCl_4_-injected mice. In addition, Sirius red staining presented the collagen deposition and fibrotic septa formed by the collagen matrix in livers of CCl_4_-treated mice (Figure 1). Those morphological changes and this collagen deposition were rarely detected in the livers of corn oil-treated mice. These results suggest that CCl_4_ promotes hepatic fibrosis in mice.

### 2.2. Dysregulated MicroRNAs’ (miRNAs) Expression in Fibrotic Livers

To investigate the miRNA expression profile in fibrotic liver, we performed miRNA array analysis on CCl_4_-induced fibrotic livers and compared them to control livers. A total of 12 miRNAs that showed more than a two-fold difference between CCl_4_-treated and control livers were filtered (*n* = 3/each group, *p* < 0.05). Among these 12 miRNAs, seven miRNAs, including mmu-miR-574-5p, mmu-miR-466i-5p, mmu-miR-342-3p, mmu-let7i-5p, mmu-miR-34a-5p, mmu-miR-188-5p and mmu-miR-5119, were upregulated, whereas five miRNAs, including mmu-miR-378a-3p, mmu-miR-202-3p, mmu-miR-378b, mmu-miR-378d and mmu-miR-212-3p, were downregulated in CCl_4_ compared to the control group (Table 1). All data of the miRNA profiles are listed in Table S1.

**Figure 2:**
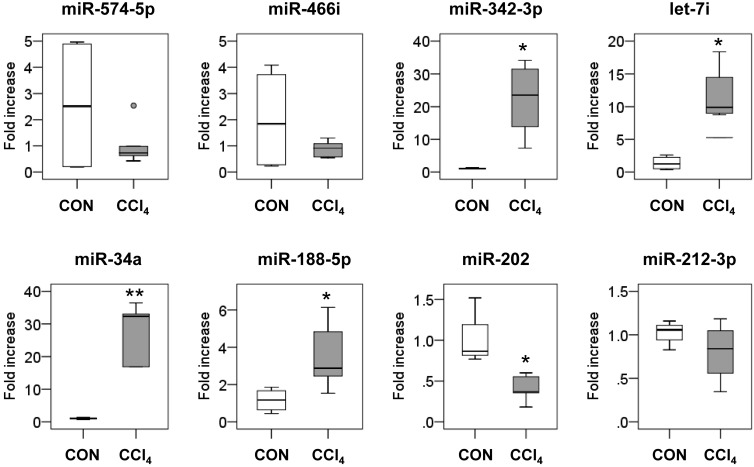
Validation of miRNA microarray results by real-time quantitative reverse transcriptional polymerase chain reaction (qRT-PCR). qRT-PCR analysis was performed to validate the expression of dysregulated miRNAs in CCl_4_-treated livers, including six upregulated miRNAs (miR-574-5p, miR-466i, miR-342-3p, let-7i, miR-34a and miR-188-5p) and two downregulated miRNAs (miR-202 and miR-212-3p) in liver tissues from corn oil-treated mice (CON) (*n* = 4) and CCl_4_-treated mice (*n* = 5). Medians and ranges are graphed (* *p* < 0.05, ** *p* < 0.005, *vs.* the CON group). A small dot indicates the maximum observation beyond the range.

To validate the data of the miRNA array, we conducted real-time quantitative reverse transcriptional polymerase chain reaction (qRT-PCR) for the expression of six upregulated miRNAs (mmu-miR-574-5p, mmu-miR-466i, mmu-miR-342-3p, mmu-let-7i, mmu-miR-34a and mmu-miR-188-5p) and five downregulated miRNAs (mmu-miR-378a-3p, mmu-miR-202, mmu-miR-378b, mmu-miR-378d and mmu-miR-212-3p) in liver tissues from the CCl_4_ group (*n* = 5) and the control group (*n* = 4). Because the reduced expression of miR-378a-3p, miR-378b and miR-378d in the fibrotic liver was demonstrated in the previous studies [17], duplicated results were not shown in the present study. Furthermore, analysis for miR-5119 was excluded in these assays, because miR-5119 was barely detected in the databases and was not conserved in humans and other species. It was confirmed that miR-342-3p, let-7i, miR-34a and miR-188-5p increased and miR-202, miR-378a-3p, miR-378b and miR-378d decreased in the CCl_4_-treated livers compared to the control livers (*p* < 0.05) (Figure 2). Thus, among the eleven miRNAs examined, the expression levels of eight miRNAs were consistent with the microarray results.

### 2.3. A Gene-Regulatory Network of miRNAs Altered in Liver Fibrosis

**Figure 3:**
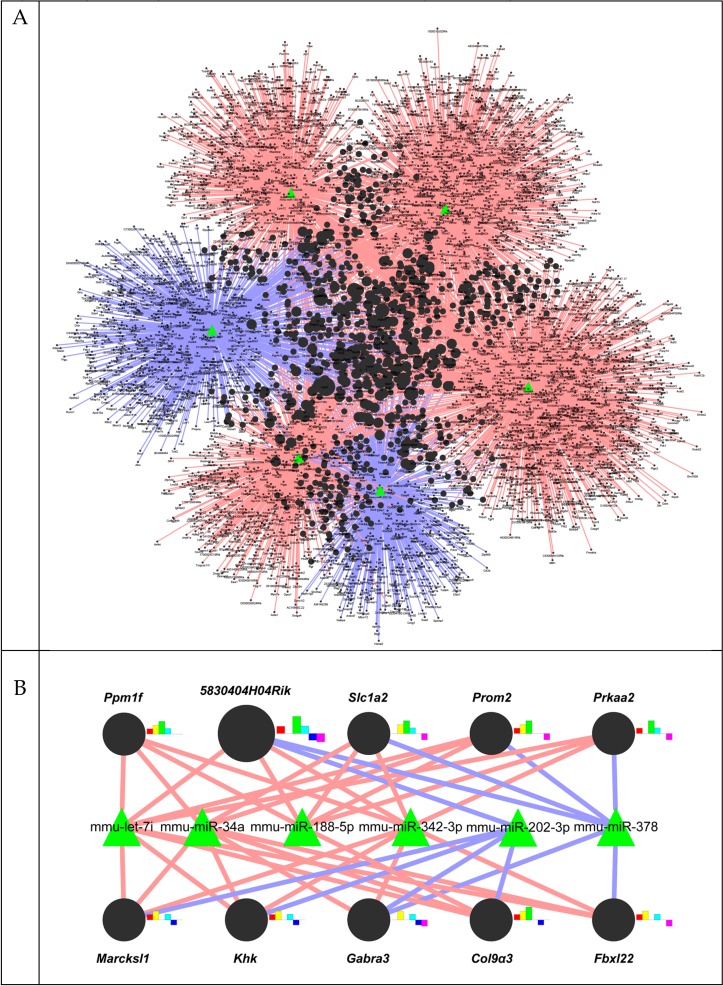
The regulatory network of miRNAs and target genes. The interaction network between miRNAs and target genes was generated by the Cytoscape program. Green-triangle and black-circle nodes indicate six differentially-expressed miRNAs and 4079 target genes, respectively. Color variations of the edges are as follows: red, upregulated miRNAs; blue, downregulated miRNAs. The size of the circle nodes indicates the numbers of associated miRNAs. The smallest node represents genes associated with one miRNA, while the largest node represents genes associated with five miRNAs. (**A**) Total network; (**B**) top 10 core networks. Bar graphs on the right side of target genes show the log change of associated miRNAs in CCl_4_-treated livers (red: let-7i, yellow: miR-34a, green: miR-188-5p, sky blue: miR-342-3p, blue: miR-202-3p, pink: miR-378).

To probe what genes were targeted by the eight miRNAs differentially expressed in liver fibrosis, as confirmed by qRT-PCR analysis, and to construct networks of these miRNAs and their target genes, we conducted bioinformatics analysis on these miRNAs using miRWalk. Because miR-378a-3p, miR-378b and miR-378d were integrated into one miR-378 family in this analysis, three individual miRNA members were counted as one miRNA. Hence, six miRNAs were investigated to find their target genes. miRWalk analysis showed that a total of 4079 genes were predicted as the target genes of these selected miRNAs (cutoff at *p* < 0.05). Figure 3A presents the whole network composed of these miRNAs and their predicted target genes. Each individual miRNA had its own target genes, forming a distinct regulatory system. Each regulatory system was linked to the core target genes regulated by more than two miRNAs, making a whole system.

**Figure 4:**
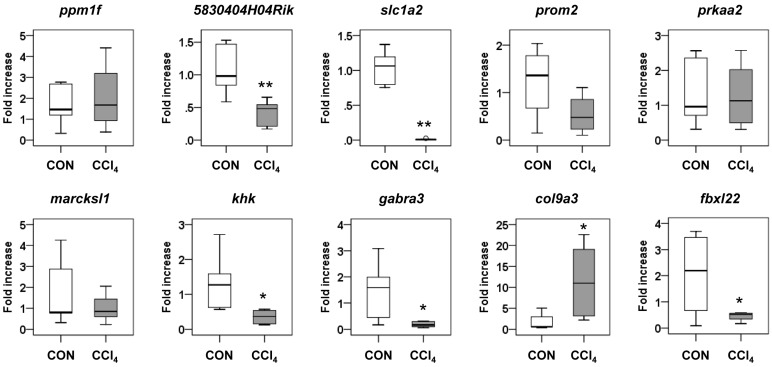
Expressions of top 10 core genes, *Ppm1f*, *5830404H04Rik*, *Slc1a2*, *Prom2*, *Prkaa2*, *Marcksl1*, *Khk*, *Gabra3*, *Col9a3* and *Fbxl22*, targeted by more than four miRNAs in liver tissues from corn oil-(CON) (*n* = 6) or CCl_4_-treated mice (*n* = 6). Medians and ranges are graphed (* *p* < 0.05, ** *p* < 0.005, *vs.* the CON group).

The top ten core target genes and their expected regulation by miRNAs are shown in Figure 3B. Interestingly, five miRNAs, including three upregulated miRNAs (let-7i, miR-188-5p and miR-342-3p) and two downregulated miRNAs (miR-202-3p and miR-378), were related to *5830404H04Rik*, a C2 calcium-dependent domain containing 2 (C2cd2). Nine other genes, protein phosphatase 1F (PP2C domain containing) (*Ppm1f*), solute carrier family 1 (glial high affinity glutamate transporter) (*Slc1a2*), prominin 2 (*Prom2*), protein kinase, AMP-activated, α 2 catalytic subunit (*Prkaa2*), MARCKS-like 1 (*Marcksl1*), ketohexokinase (*Khk*), γ-aminobutyric acid (GABA) A receptor, subunit α 3 (*Gabra3*), collagen, type IX, α 3 (*Col9a3*) and F-box and leucine-rich repeat protein 22 (*Fbxl22*), were related to the different combinations of four up- or downregulated miRNAs. A list of all predicted target genes is shown in Table S2. To examine whether the miRNAs influenced the expression of their predicted target genes, we conducted qRT-PCR analysis for these genes. The RNA levels of *5830404H04Rik*, *Slc1a2*, *Khk*, *Gabra3*, *Col9a3* and *Fbxl22* were significantly changed in the CCl_4_ group compared to the control group (Figure 4). These data indicated that dysregulated miRNAs might modulate the expression of their target genes during liver fibrogenesis.

### 2.4. Functional Analysis of the Predicted Target Genes

**Figure 5:**
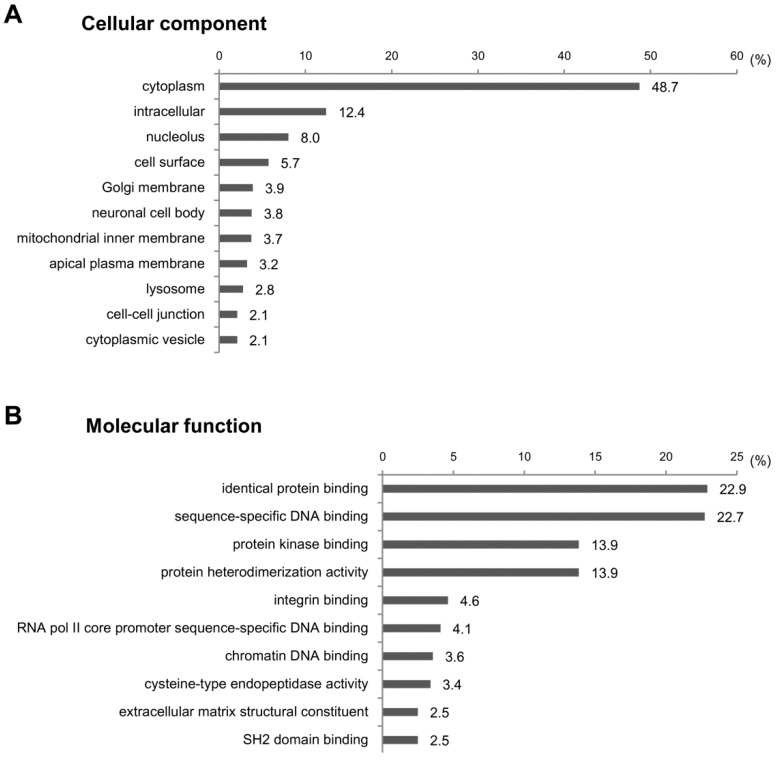

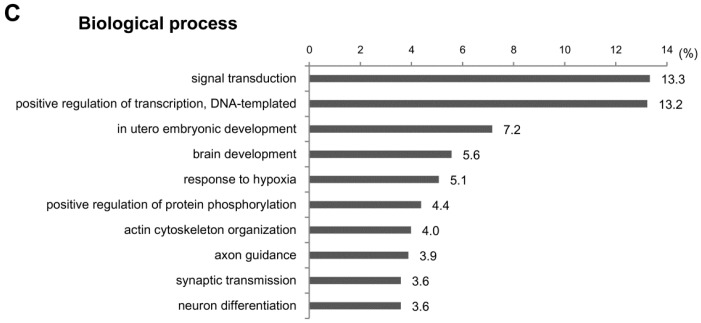
GO analysis of predicted target genes of dysregulated miRNAs in CCl_4_-induced liver fibrosis. GO enrichment analysis was conducted to investigate the functions of target genes of differentially-expressed miRNAs in CCl_4_-treated livers. A total of 63 terms were identified based on a *p*-value of <0.001. The top 10 GO terms and percentage of target genes are displayed in the graph. All data from the GO analyses on miRNA-target genes are listed in Table S3. (**A**) Cellular component; (**B**) molecular function; (**C**) biological process.

To investigate the functions of the target genes predicted above, we performed GO analysis for the cellular components, molecular functions and biological processes. We cut off the GO enrichment data at *p* < 0.001 and calculated the percentages of target genes involved in subcategories (Figure 5). In cellular component analysis, we found that about half of target genes were located in the cytoplasm (48.7%) (Figure 5A). Other target genes were scattered in various subcellular loci, including the intracellular (12.4%), nucleolus (8%) and cell surface (5.7%). In the molecular function assay, most target genes were related to protein-protein or protein-DNA bindings, such as the identical protein binding (22.9%), sequence-specific DNA binding (22.7%), protein kinase binding (13.9%) and protein heterodimerization activity (13.9%) (Figure 5B). In line with the fact that the protein-protein interactions are involved in the signal transduction controlling gene expression [18], the signal transduction (13.3%) and the DNA-templated positive control of transcription (13.2%) were the largest categories in biological process (Figure 5C). All data from the GO analyses on miRNA-target genes are listed in Table S3.

To further understand the biological functions of the predicted target genes in liver fibrosis, we performed KEGG pathway analyses, which provided more detailed information about the signaling pathways in which the target genes were involved. The key signaling pathways regulated by six miRNAs were selected by KEGG enrichment at *p* < 0.05 and are listed in Table 2. Interestingly, 24 of the 72 total pathways were directly related to metabolism, including metabolic pathways (25.3%), other types of *O*-glycan biosynthesis (48.4%), sphingolipid metabolism (41.7%), glycosaminoglycan biosynthesis (keratan sulfate (60%), lysine degradation (39.2%), *N*-glycan biosynthesis (38.8%), fatty acid biosynthesis (57.1%), ovarian steroidogenesis (36.2%), fatty acid degradation (36.7%), glycosphingolipid biosynthesis), lacto and neolacto series (42.3%), glycerolipid metabolism (34.5%), fatty acid metabolism (34%), valine, leucine and isoleucine degradation (33.3%), glycolysis/gluconeogenesis (31.8%), amino sugar and nucleotide sugar metabolism (34%), melanogenesis (29%), thiamine metabolism (75%), valine, leucine and isoleucine biosynthesis (75%), tryptophan metabolism (32.6%), purine metabolism (25.7%), thyroid hormone synthesis (29.6%), glycerophospholipid metabolism (28.3%), citrate cycle (tricarboxylic acid (TCA) cycle) (34.4%) and glycosaminoglycan degradation (38.1%). Additionally, several signaling pathways involved in cellular metabolic processes were included in the significantly-annotated pathways by KEGG pathway analysis. For example, the hedgehog (Hh) signaling pathway (34.7%) and tumor necrosis factor (TNF) signaling pathway (29.4%) play the critical roles in regulating metabolism of HSCs [19] and adipocytes [20], respectively. The percentages in parenthesis indicate the proportion of target genes in total genes associated with the pathway. All data from the KEGG pathway analysis of the target genes are listed in Table S4.

### 2.5. Interaction of miRNA Target Involved in Hedgehog Signaling

**Figure 6:**
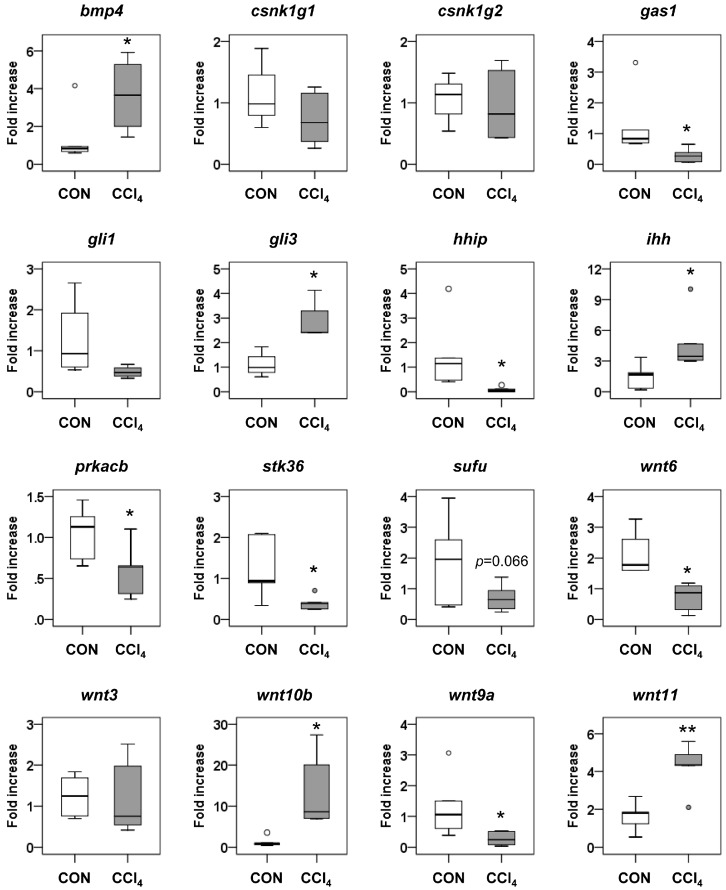
Expressions of target genes involved in the Hh signaling pathway. qRT-PCR analyses on the genes involved in Hh signaling, including *Bmp4*, *Csnk1g1*, *Csnk1g2*, *Gas1*, *Gli1*, *Gli3*, *Hhip*, *Ihh*, *Prkacb*, *Stk36*, *Sufu*, *Wnt6*, *Wnt3*, *Wnt10b*, *Wnt9a* and *Wnt11*, were conducted in liver tissues from corn oil-treated mice (CON) (*n* = 6) and CCl_4_-treated mice (*n* = 6). Medians and ranges are graphed (* *p* < 0.05, ** *p* < 0.005, *vs.* the CON group). Small dots indicate the maximum or minimum observations beyond the range.

Among the significantly-enriched 72 pathways in the KEGG pathway analysis, Hh signaling is in our interest because it contributes to the liver fibrosis by stimulating the activation of HSCs [19,21]. To further understand how miRNAs regulate the genes involved in Hh signaling, we examined the expression of target genes involved in the Hh signaling pathway by qRT-PCR analysis. In Table 3, we summarized the Hh signaling-related putative target genes, such as bone morphogenetic protein 4 (*Bmp4*), casein kinase 1 γ 1 (*Csnk1g1*), *Csnk1g2*, growth arrest specific 1 (*Gas1*), GLI-Krüppel family member 1 (*Gli1*), *Gli3*, hedgehog-interacting protein (*Hhip*), Indian Hh (*Ihh*), cyclic adenosine monophosphate (cAMP)-dependent protein kinase catalytic subunit β (*Prkacb*), serine/threonine kinase 36 (*Stk36*), suppressor of fused homolog (*Sufu*) and members of the wingless-type mouse mammary tumor virus (MMTV) integration site family (*Wnt6*, *Wnt3*, *Wnt3a*, *Wnt10b*, *Wnt9a* and *Wnt11)*. Among the total of 17 genes, the expression of eleven genes was significantly changed in livers of the CCl_4_-treated mice compared to the corn oil-treated mice (*p* < 0.05) (Figure 6). The mRNA expression of *Wnt3a* was non-detectable in both corn oil- and CCl_4_-treated livers. Two Hh-activator genes, *bmp4* and *gli3*, targeted by miR-378, were upregulated as miR-378 was downregulated in the fibrotic liver (Table 1 and Figure 6). *Hhip*, a well-established Hh inhibitor [22,23], was analyzed to be targeted by miR-342-3p, and its expression was reduced with the increase of miR-342-3p during liver fibrosis (Table 1, Figure 2 and Figure 6). In addition, the RNA level of *Gas1*, *Prkacb*, *Stk36* and *Wnt9a* significantly decreased as the expression of their regulatory miRNAs, miR-188-5p for *Gas1*, miR-34a for *Prkacb*, let-7i for *Stk36* and let-7i and miR-342-3p for *Wnt9a*, increased in the CCl_4_ group compared to the control group. The expression of these target genes was inversely correlated with the expression of their regulatory miRNAs, showing the inhibitory effect of miRNAs on their target genes. On the other hand, the expression of *Ihh*, *Wnt10b* and *Wnt11* was elevated with the increase of their regulatory miRNAs, miR-34a, miR-342-3p and miR-188-5p, respectively, and the level of *Wnt6* was reduced with the decrease of its regulator miRNA, miR-378, in the CCl_4_ group, implying that other regulatory factors might be additionally involved in the expression of these genes. These results suggest that the miRNAs influence the liver fibrosis by altering the expression of target genes associated with Hh signaling.

## 3. Discussion

miRNAs are known to be involved in many biological processes, and their dysregulation has been reported in several diseases. miRNAs are more stable than messenger RNA and are available for quantification in various body fluids, such as serum, plasma, urine and saliva [24,25]. Thus, miRNAs are expected to be novel biomarkers. In addition, successful in vivo manipulation of certain miRNA using oligonucleotide transfection and a viral expression system suggests that miRNAs are potential therapeutic agents [26,27]. However, it is not fully understood what miRNAs are altered and what and how biological procedures are affected by miRNAs during liver fibrogenesis.

In the present study, we analyzed the liver of mice treated for 10 weeks with CCl_4_ by microarray. To investigate the distinct signaling pathways involved in CCl_4_-induced liver fibrosis, we also performed genome-wide GO and in-depth KEGG pathway enrichment analysis for the target genes of miRNAs. We found that eight miRNAs (four upregulated and four downregulated) were significantly dysregulated in CCl_4_-treated livers compared to corn oil-treated livers (*p* < 0.05), and a total of 4079 target genes were predicted for these miRNAs. GO enrichment analysis revealed that most of the putative target genes were located in the cytoplasm where most cellular biochemical reactions occur. With respect to molecular functions and biological processes, most of the miRNA target genes were involved in signal transduction, which is a series of biochemical events in response to extracellular signals and mediates the metabolism and gene expression in the cells. For example, miR-26a targeting several key regulators of hepatic metabolism and insulin signaling, such as GSK3β, PKCδ/θ, ACSL3/4, PCK1 and TCF7L2, ameliorates the obesity-induced metabolic complications by improving the insulin sensitivity and decreasing both of the hepatic glucose production and the fatty acid synthesis in mice fed with a high-fat diet [28].

In the KEGG pathway enrichment analysis for assessing the biological functions of putative target genes, we sorted out the 72 total signaling pathways that might be critical in the progression of liver fibrosis (*p* < 0.05). We found several pathways that were known to be involved in liver fibrosis, such as the AMPK signaling pathway (36.4%) [29], the FoxO signaling pathway (31.1%) [30], the Notch signaling pathway (38.8%) [31], the mitogen-activated protein kinase (MAPK) signaling pathway (26.9%) [32], the estrogen signaling pathway (30.6%) [33], the p53 signaling pathway (32.4%) [34], the hedgehog signaling pathway (34.7.5%) [35], the mechanistic target of rapamycin (mTOR) signaling pathway (32.8%) [36], hepatitis B (27.6%) [37], the PI3K-Akt signaling pathway (24.5%) [38], cAMP signaling pathway (25.8%) [39], the TGF-β signaling pathway (29.3%) [40] and the hypoxia-inducible factor (HIF)-1 signaling pathway (27%) [41]. In addition, a main biological process involving various metabolic pathways was found in this analysis.

Metabolic pathways are closely associated with liver functions, because the hepatocytes occupying over 80% of the liver mass are responsible for carbohydrate, lipid and protein metabolism. Metabolic syndromes, such as obesity, diabetes, hyperdyslipidemia, hypercholesterolemia and insulin resistance, are related to nonalcoholic fatty liver disease, capable of ongoing hepatic fibrogenesis [42]. Recently, altered protein glycosylation has been shown to regulate the activation of HSCs [43] and the pathogenesis of various liver diseases [44]. Glycoproteins, such as fibronectin, laminin and hyaluronic acid, are overexpressed in fibrotic livers [45]. In addition, the oligonucleotide microarray for glycan-related genes and the lectin (glycan-recognizing protein) microarray for structure profiling of glycan reported the altered expression of glycan-related genes and changed glycan patterns on the surface of hepatocytes in the fibrotic livers of CCl_4_-treated mice [46]. In line with these findings, glycan-associated metabolic pathways, including the other types of *O*-glycan biosynthesis (48.4%), glycosaminoglycan biosynthesis (keratin sulfate (60%)), *N*-glycan biosynthesis (38.8%), glycosaminoglycan degradation (38.1%) and proteoglycans in cancer (24.9%), were highly enriched in the present study.

Recent studies demonstrate that Hh signaling plays the essential roles in liver fibrosis by controlling the metabolism of HSCs. Hh signaling induces the activation of HSCs by increasing the expression of genes involved in glycolysis, whereas the suppression of the Hh pathway in activated HSCs reduces the number of glycolysis-occurring HSCs and the degree of liver fibrosis in mice [19]. Furthermore, curcumin, the polyphenolic chemical from turmeric, reduces the expression of glycolysis-regulatory genes by inhibiting the Hh signaling pathway in HSCs of rat fibrotic liver [47]. These findings suggest that the miRNAs targeting Hh signaling, among differentially-expressed miRNAs in liver fibrosis, influence the HSC metabolic pathways and activation by regulating Hh signaling. In the current study, the Hh-related genes, including *Bmp4*, *Gas1*, *Gli3*, *Hhip*, *Ihh*, *Prkacb*, *Stk36*, *Wnt6*, *Wnt10b*, *Wnt9a* and *Wnt11*, were dysregulated in our murine model of CCl_4_-induced liver fibrosis. Hhip is a well-known repressor of Hh ligands (Sonic Hh, Indian Hh, Desert Hh) [48,49] and known to be downregulated in the injured liver [50]. As the expression of *Hhip* was lowered, its putative regulator miR-342-3p and the RNA level of *Ihh* and *Gli3* were significantly elevated in the CCl_4_ group compared to the control group (*p* < 0.05). These findings imply that the activation of Ihh-mediated Hh signaling may be promoted by miR-342-3p-mediated inhibition of Hhip during liver fibrosis. *Bmp4* was reported to be induced by Sonic Hh and Ihh [51,52], and its expression was significantly elevated in the fibrotic livers of rat with bile duct ligation and an in vitro-cultured rat HSC line [53]. In line with these findings, the RNA level of *Bmp4* was greatly higher in CCl_4_-treated fibrotic liver than corn oil-treated liver of mice in the present study. Although four other Hh-related genes, including *Gas1*, *Prkacb*, *Stk36* and *Wnt9a*, were significantly downregulated with the simultaneous upregulation of their regulator miRNAs in the CCl_4_ group compared to the control group, the roles of these Hh-related genes in liver fibrosis is poorly understood. Further studies are needed to elucidate their function in liver. Consistent with our data, *Wnt10b* was reported to be elevated in primary rat HSCs during activation [54]. Although the function of *Wnt11* and *Wnt6* in liver fibrosis was unknown, *Wnt11* was shown to enhance the expression of mesenchymal markers, such as Zeb1, Snail1, Pai1 and α-SMA, in response to the TGF-β signaling in renal fibrosis [55], and *Wnt6* was reported to be downregulated during renal fibrogenesis [56]. *Wnt10b*, a putative target gene of miR-342-3p, and *Wnt11* targeted by miR-188-5p were upregulated in the fibrotic liver, although the expression of miR-342-3p and miR-188-5p were elevated. In addition, the expression of w*nt6* targeted by miR-378 decreased, despite the reduction of miR-378 in CCl_4_-treated liver. These results suggest that other factors might be involved in regulating the expression of these genes, rather than the miRNA-induced gene silencing. It is also possible that the miRNAs positively regulate the expression of *Wnt10b*, *Wnt11* and *Wnt6*, because miRNAs are known to be able to upregulate the expression of their target genes depending on the types of cells, cell culture condition and association with other regulatory elements [57,58]. Therefore, additional studies are further required to understand what kinds of miRNAs positively or negatively regulate the expression of their putative target genes, and consequentially, how they influence cellular events during liver fibrosis.

In the KEGG analysis presenting the relation between miRNAs and their target genes, a total of 843 putative target genes were predicted to be involved in the top 72 pathways associated with CCl_4_-induced hepatic fibrosis. Among these predicted targets, 680 genes were each individually connected with a single dysregulated miRNA, whereas 163 genes were related to more than two miRNAs. *Prkaa2*, one of the core target genes (Figure 3B) related to miR-378, miR-342-3p, miR-188-5p and let-7i, seems to be involved in the FoxO signaling pathway, mTOR signaling pathway, PI3K-Akt signaling pathway, AMPK signaling pathway, insulin signaling pathway and the adipocytokine signaling pathway. Of the putative target genes, 231 target genes were predicted to be modulated by miR-342-3p, which was reported to control lipogenesis and cholesterogenesis by targeting SREBP-1, a key transcription factor for lipogenesis-related genes, such as fatty acid synthase (FASN) and 3-hydroxy-3-methylglutaryl-CoA reductase (HMGCR). In addition, the level of miR-342-3p was reported to be higher in liver cirrhosis and NASH than chronic hepatitis and nonalcoholic fatty liver, respectively, although its function in cirrhosis and NASH was not investigated [59,60]. miR-34a was predicted to be connected with 245 target genes. miR-34a, which is a member of the miR-34 family composed of miR-34a, -34b and -34c, is well known as a tumor suppressor in various kinds of cancers, including liver cancer [61]. During liver regeneration after partial hepatectomy, miR-34a was highly upregulated [62]. In addition, matrix metallopeptidase 9 (MMP9), a marker of activated HSC, was upregulated in human hepatocytes transfected with miR-34a precursor, and the level of miR-34a increased in ethanol-exposed hepatocyte and liver followed by upregulation of the MMP9 in mice [63]. Taken together, those results indicate that miR-34a is associated with liver fibrosis.

Among the downregulated miRNAs, the expression of miR-378 was validated in both the fibrotic livers of CCl_4_-treated mice and in the activated HSCs in our previous study [17]. We demonstrated that miR-378 promoted the inactivation of HSCs by targeting Gli3, a transcriptional factor of Hh signaling. This reduced expression of miR-378 and induced expression of miR-34a-5p were also shown in the fibrotic liver of dimethylnitrosamine (DMN)-treated rats [64] and methionine/choline-deficient (MCD)-dieted mice [65]. In addition, miR-202 was shown to be a tumor suppressor that inhibited cell proliferation by targeting Gli1, a transcription factor of the Hh signaling pathway, in gastric cancer [66]. In the current studies, miR-202 was predicted to target Col9α3, a fibrillar collagen, increased in fibrotic liver. These findings suggest the potential of miR-202 as an anti-fibrotic factor in the liver.

We reported eight miRNAs differentially expressed in the fibrotic liver, whereas other groups found more than eight miRNAs showing the change of expression in the fibrotic liver [64,65,67]. There are several factors influencing the data, such as the method of microarray analysis, mouse strain, chemical, period of chemical or injury treatment [17]. The SurePrint G3 Mouse miRNA Microarray (Agilent, Santa Clara, CA, USA) that covered 1247 unique mouse miRNA targets using a more updated version (miRBase v19.0 [68]) was employed to analyze our sample. Hence, our microarray data analyzed by the more advanced version provided the updated miRNA expression profiles in chronically-damaged liver with fibrosis. In addition, the expression change of eight miRNAs among 12 miRNAs was validated through qRT-PCR. As described above, up- or downregulation of miR-378, miR-34a-5p and miR-202 was reported by other independent studies [64,65,66].

## 4. Materials and Methods

### 4.1. Experimental Animal Model

Animal models of liver fibrosis were constructed as previously described [17,69]. Six-week-old male C57BL/6 mice were purchased from Hyochang (Dae-gu, Korea), maintained under a twelve-hour light/dark cycle and allowed free access to food and water. After one week for adaptation, mice received intraperitoneal injection of 0.6 mL/kg body weight of carbon-tetrachloride (CCl_4_, Sigma-Aldrich, St. Louis, MO, USA) dissolved in corn oil, twice a week for ten weeks, in order to induce liver fibrosis. Mice having the same volume of corn oil were used for the control group (*n* = 6/each group). At forty-eight hours after the last injection, mice were sacrificed, and liver tissues were collected. This study was carried out in strict accordance with the recommendations in the Guide for the Care and Use of Laboratory Animals of the National Institutes of Health (NIH, New York, NY, USA). All animal care and experiments were approved by the Pusan National University Institutional Animal Care and Use Committee (PNU-IACUC).

### 4.2. Hematoxylin-Eosin and Sirius Red Staining

Liver specimens were fixed in 10% neutral buffered formalin (NBF, Sigma-Aldrich), embedded in paraffin and cut into 4-µm sections. To perform the H&E and Sirius red staining to examine hepatic morphology and fibrosis, respectively, specimens were deparaffinized, hydrated and stained by standard methods.

### 4.3. RNA Isolation and MicroRNA Microarray

Total RNA, including miRNA, was purified from liver tissues by using TRIzol Reagent (Ambion, Thermo Fisher Scientific, Waltham, MA, USA) according to the manufacturer’s instruction. RNA quantity and quality were determined using a NanoDrop (Thermo Fisher Scientific Waltham, MA, USA) and Bioanalyzer (Agilent, Santa Clara, CA, USA). Desalted Cy3-labeled RNA was prepared from 100 ng of total RNA by using Agilent’s miRNA Complete Labeling and Hyb Kit (Agilent, Santa Clara, CA, USA) and then hybridized to the Agilent miRNA expression microarray according to the protocols provided by the manufacturer. The SurePrint G3 Mouse miRNA Microarray, Release 19.0, 8 × 60 K (Agilent, Inc., Santa Clara, CA, USA), platform was used for miRNA profiling, and raw data were extracted using the software provided by Agilent Feature Extraction Software (v11.0.1.1). For microarray analysis, raw data were firstly filtered by flag signal as the detection in all samples. Filtered raw data were processed using the limma Bioconductor package [70] in the R statistical environment [71]. After the quantile normalization of data, differentially-expressed miRNAs were identified with *p* < 0.05 and fold changes >2. The microarray data have been deposited in the NCBI GEO database under the accession code GSE77271.

### 4.4. Real-Time Quantitative Reverse Transcriptional Polymerase Chain Reaction Analysis

Template cDNA was synthesized from total RNA using the miScript Reverse Transcriptase Kit (Qiagen, Hilden, Germany) or the SupersScript First-strand Synthesis System (Invitrogen, Thermo Fisher Scientific, Waltham, MA, USA) according to the manufacturer’s protocol. Then, real-time PCR was performed using the miScript SYBR Green PCR Kit (Qiagen) or Power SYBR Green Master Mix (Applied Biosystem, Thermo Fisher Scientific, Waltham, MA, USA) on the manufacturer’s specification (Mastercycler Real-Time PCR machine, Eppendorf, Hamburg, Germany). All reactions were in triplicate, and data were analyzed according to the ∆∆*C*_t_ method. U1A small nuclear RNA (RNU1A) for miRNA and 40S ribosomal protein S9 mRNA for mRNA were used for normalization of the expression level. The sequences of miRNA primers used in this study are as follows: mmu-miR-574-5p, TGAGTGTGTGTGTGTGAGTGTGT; mmu-miR-466i, TGTGTGTGTGTGTGTGTGTG; mmu-miR-342-3p, TCTCACACAGAAATCGCACCCGT; mmu-let-7i-5p, TGAGGTAGTAGTTTGTGCTGTT; mmu-miR-34a-5p, TGGCAGTGTCTTAGCTGGTTGT; mmu-miR-188-5p, CATCCCTTGCATGGTGGAGGG; mmu-miR-202-3p, AGAGGTATAGCGCATGGGAAGA; mmu-miR-212-3p, TAACAGTCTCCAGTCACGGCCA; U1A snRNA, CGACTGCATAATTTGTGGTAGTGG. These primers were used in combination with the miScript Universal primer (Qiagen). The sequences of mRNA primers used in this study are listed in Table 4.

### 4.5. Statistical Analysis

Results of qRT-PCR analysis are shown as the mean ± standard error of mean (SEM) among individuals of the same group. Statistical significances between control and CCl_4_-treated groups were analyzed by the unpaired two-sample Student’s *t*-test, and *p* < 0.05 (one-tailed) was considered as significantly different.

### 4.6. MicroRNA Target Prediction

The miRWalk database [72] was used to identify target genes of 6 differentially-expressed miRNAs. In this analysis, genes with *p* < 0.05 were regarded as predicted and validated targets. To construct the overall network between miRNAs and target genes, we visualize interactions using the Cytoscape software [73]. In a network, green triangles represent differentially-expressed miRNAs, and black circles represent target genes. The color variations of edges are as follows: red, upregulated miRNAs; blue, downregulated miRNAs. Furthermore, the size of circles reflects the number of interactions with miRNAs.

### 4.7. Gene Ontology and Kyoto Encyclopedia of Genes and Genomes Enrichment Analyses

To investigate the biological functions of potential target genes controlled by hepatic fibrosis-associated miRNAs, we attempted to conduct GO enrichment analysis. To accomplish this, we downloaded GO annotation data for *Mus musculus* from the UniProt database [74] and matched to target genes via the python language script. In addition, KEGG enrichment analysis was carried out using total pathways and modules’ data. These data were downloaded using the WGET program that retrieves contents from web sites [75] and matched to target genes in the same way as for GO enrichment analysis. Finally, the results of GO and KEGG enrichment analysis were ranked with statistical significance by calculating their *p*-values based on hypergeometric distribution with *p* < 0.001 and *p* < 0.05 considered to indicate significance, respectively [76].

## 5. Conclusions

In conclusion, we identified a total of eight miRNAs differentially expressed in fibrotic livers of CCl_4_-treated mice compared to the control livers of corn oil-treated mice. We first performed the bioinformatics analysis for these eight miRNAs, including target prediction, using miRWalk, GO analysis and KEGG pathway enrichment analysis. This approach demonstrates that enriched GO and pathways are consistent with well-known and associated signaling pathways involved in liver fibrosis. In addition, it showed that the dynamic interactions of various signaling pathways contributed to hepatic fibrogenesis and that these complicated networks were regulated by miRNAs. Although further study is needed to verify the potential roles of these miRNAs in liver, they may help to develop novel diagnostic biomarkers and therapeutic targets in chronic liver diseases with fibrosis.

## Figures and Tables

**Table 1:** Dysregulated microRNAs (miRNAs) in carbon-tetrachloride (CCl_4_)-induced liver fibrosis (>2-fold, *p* < 0.05).

Probe ID	Log2 Change (CCl_4_/Corn Oil)	Fold Change (CCl_4_/Corn Oil)	Adjust *p*-Value
Upregulated miRNAs			
mmu-miR-574-5p	3.625210638	12.33949	0.000259147
mmu-miR-466i-5p	3.084363793	8.481761	0.000830411
mmu-miR-342-3p	1.180557713	2.266644	0.003273323
mmu-let-7i-5p	1.136496066	2.198464	0.016247731
mmu-miR-34a-5p	1.871455739	3.659016	0.032876548
mmu-miR-188-5p	2.700779126	6.501529	0.042779303
mmu-miR-5119	1.048052536	2.067737	0.042779303
Downregulated miRNAs			
mmu-miR-378a-3p	−1.338779005	0.395355	0.00162313
mmu-miR-202-3p	−1.079114551	0.473319	0.003273323
mmu-miR-378b	−1.360233472	0.389519	0.003273323
mmu-miR-378d	−1.424843795	0.37246	0.003273323
mmu-miR-212-3p	−1.132709203	0.456058	0.018962762

**Table 2:** Significantly-enriched top 40 pathways of the target genes of differently-expressed miRNAs in CCl_4_-induced liver fibrosis (*p* < 0.05).

KEGG ID	Pathway	*p*-Value	KEGG ID	Pathway	*p*-Value
mmu01100	Metabolic pathways	8.71 × 10^−8^	mmu04020	Calcium signaling pathway	3.75 × 10^−3^
mmu04152	AMPK signaling pathway	6.75 × 10^−6^	mmu00071	Fatty acid degradation	4.23 × 10^−3^
mmu04920	Adipocytokine signaling pathway	4.78 × 10^−5^	mmu04022	cGMP-PKG signaling pathway	4.62 × 10^−3^
mmu05200	Pathways in cancer	1.90 × 10^−4^	mmu04915	Estrogen signaling pathway	6.78 × 10^−3^
mmu00514	Other types of *O*-glycan biosynthesis	3.13 × 10^−4^	mmu00601	Glycosphingolipid biosynthesis: lacto and neolacto series	7.17 × 10^−3^
mmu00600	Sphingolipid metabolism	4.13 × 10^−4^	mmu00561	Glycerolipid metabolism	7.21 × 10^−3^
mmu00533	Glycosaminoglycan biosynthesis: keratan sulfate	7.12 × 10^−4^	mmu04115	p53 signaling pathway	9.55 × 10^−3^
mmu04910	Insulin signaling pathway	9.09 × 10^−4^	mmu04666	Fc γ R-mediated phagocytosis	9.57 × 10^−3^
mmu00310	Lysine degradation	1.04 × 10^−3^	mmu04014	Ras signaling pathway	1.00 × 10^−2^
mmu04068	FoxO signaling pathway	1.08 × 10^−3^	mmu04725	Cholinergic synapse	1.01 × 10^−2^
mmu04974	Protein digestion and absorption	1.34 × 10^−3^	mmu04340	Hedgehog signaling pathway	1.02 × 10^−2^
mmu04210	Apoptosis	1.53 × 10^−3^	mmu04668	TNF signaling pathway	1.03 × 10^−2^
mmu00510	*N*-Glycan biosynthesis	1.62 × 10^−3^	mmu04660	T cell receptor signaling pathway	1.05 × 10^−2^
mmu04330	Notch signaling pathway	1.62 × 10^−3^	mmu04150	mTOR signaling pathway	1.12 × 10^−2^
mmu04080	Neuroactive ligand-receptor interaction	1.83 × 10^−3^	mmu04142	Lysosome	1.25 × 10^−2^
mmu04611	Platelet activation	2.06 × 10^−3^	mmu04550	Signaling pathways regulating pluripotency of stem cells	1.26 × 10^−2^
mmu00061	Fatty acid biosynthesis	2.21 × 10^−3^	mmu01212	Fatty acid metabolism	1.27 × 10^−2^
mmu04913	Ovarian steroidogenesis	2.56 × 10^−3^	mmu05340	Primary immunodeficiency	1.28 × 10^−2^
mmu04360	Axon guidance	2.84 × 10^−3^	mmu00280	Valine, leucine and isoleucine degradation	1.31 × 10^−2^
mmu04010	MAPK signaling pathway	3.34 × 10^−3^	mmu00010	Glycolysis/gluconeogenesis	1.36 × 10^−2^

MAPK, mitogen-activated protein kinase; PKG, protein kinase G; TNF, tumor necrosis factor; mTOR, mechanistic target of rapamycin.

**Table 3:** Putative target genes involved in the Hedgehog signaling pathway.

RefSeq	Gene Name	Targeting miRNA	Other Involved Pathways
NM_007554	Bmp4	mmu-miR-378	TGF-β signaling pathway, signaling pathways regulating pluripotency of stem cells, pathways in cancer, basal cell carcinoma
NM_173185	Csnk1g1	mmu-miR-202-3p	-
NM_134002	Csnk1g2	mmu-miR-378	-
NM_008086	Gas1	mmu-miR-188-5p	-
NM_010296	Gli1	mmu-miR-188-5p	cAMP signaling pathway, pathways in cancer, basal cell carcinoma
NM_008130	Gli3	mmu-miR-378	
NM_020259	Hhip	mmu-miR-342-3p	
NM_010544	Ihh	mmu-miR-34a	-
NM_011100	Prkacb	mmu-miR-34a	MAPK signaling pathway, Ras signaling pathway, calcium signaling pathway, cAMP signaling pathway, apoptosis, adrenergic signaling in cardiomyocytes, platelet activation, cholinergic synapse, insulin signaling pathway, ovarian steroidogenesis, estrogen signaling pathway, melanogenesis, thyroid hormone synthesis, vasopressin-regulated water reabsorption, salivary secretion, bile secretion, pathways in cancer, proteoglycans in cancer, dilated cardiomyopathy
NM_175031	Stk36	mmu-let-7i	Pathways in cancer, basal cell carcinoma
NM_015752	Sufu	mmu-miR-34a	
NM_009526	Wnt6	mmu-miR-378	Signaling pathways regulating pluripotency of stem cells, melanogenesis, pathways in cancer, proteoglycans in cancer, basal cell carcinoma
NM_009522	Wnt3a	mmu-let-7i	
NM_009521	Wnt3	mmu-miR-342-3p	
NM_011718	Wnt10b		
NM_139298	Wnt9a	mmu-let-7i, mmu-miR-342-3p	
NM_009519	Wnt11	mmu-miR-188-5p	

TGF, transforming growth factor; cAMP, cyclic adenosine monophosphate; MAPK, mitogen-activated protein kinase.

**Table 4:** Primer sequences of mRNA used for qRT-PCR analysis.

Gene	Forward (5’ to 3’)	Reverse (5’ to 3’)
*Ppm1f*	GCTTCTTCAACTGCCTTTGG	CCATGACCATCAAACACAGC
*5830404H04Rik*	GCTGGCTCATTTTCTTCAGG	CGGAAGAAAAGCACCATCAT
*Slc1a2*	TCTGAGGAGGCCAATACCAC	TTCATCCCGTCCTTGAACTC
*Prom2*	GTGTGACATGATGGCTGACC	ACCCTGGGGATATGGAAAAG
*Pkaa2*	CGGCGCCTTTCCTTGAATAT	GGCCTGTTCCTCACGGTATTA
*Marcksl1*	GGCAGCCAGAGCTCTAAGG	TCACGTGGCCATTCTCCT
*Khk*	AGCCTCATGGAAGAGAAGCA	GGAGGTCATCCAGGACAAAA
*Gabra3*	ATGTGGCACTTTTATGTGACCA	CCCCAGGTTCTTGTCGTCTTG
*Col9a3*	CAAGGATGGCATTGATGGAG	CAGACCATCTACACCAGGCAGT
*Fbxl22*	ATGGGCAAGCAGGTTAAATG	CACGCGAACAGAACAGAAAA
*Bmp4*	GAGGAGGAGGAAGAGCAGAG	TGGGATGTTCTCCAGATGTT
*Csnk1g1*	CAGTGGTTTTCACCTTCTGG	AGGCCTTCACCTGCACTG
*Csnk1g2*	TACTACTTCGGCCCTTGTGG	GGCTTCACGTCACGGTAGAT
*Gas1*	TAAATTGCTTGTGACCACTG	GAGCATTACCGATGGATAGA
*Gli1*	TGTGTGAGCAAGAAGGTTGC	ATGGCTTCTCATTGGAGTGG
*Gli3*	GCAACCTCACTCTGCAACAA	CCTTGTGCCTCCATTTTGAT
*Hhip*	GGTCACATCTTGGGATTTGG	TCCATTGTGAGTCTGGGTCA
*Ihh*	CAATCCCGACATCATCTTCA	AGTTCAGACGGTCCTTGCAG
*Prkacb*	TCTTTCCTGCGTCATCAGTG	AAGGGAGCACTGGTCAGAGA
*Stk36*	CCAGGAGAGTAGCAGCATCC	TGTCTGCTCGGAATCATCTG
*Sufu*	TCCAGGTTACCGCTATCGTC	TCCACTGTTGGGCTGAATGT
*Wnt6*	TGTCAGTTCCAGTTCCGTTTCC	GCTGCGGTGATTGCAAACA
*Wnt3a*	GGCTCCTCTCGGATACCTCT	GGGCATGATCTCCACGTAGT
*Wnt3*	CAAGCACAACAATGAAGCAGG	TCGGGACTCACGGTGTTTC
*Wnt10b*	ACGACATGGACTTCGGAGAGAAGT	CATTCTCGCCTGGATGTCCC
*Wnt9a*	GGCACAGGGTTACAAACAAC	GGACAGAGGCAACTGAGAAA
*Wnt11*	ATGTGCGGACAACCTCAGCTA	CGCATCAGTTTATTGGCTTGG

## Supplementary Materials

Supplementary materials can be found at http://www.mdpi.com/1422-0067/17/6/961/s1.

## Abbreviations

HBV/HCV	hepatitis B/C virus
NASH	non-alcoholic steatohepatitis
miRNA	microRNAs
HSC	hepatic stellate cells
TGF	transforming growth factor
CCl_4_	carbon-tetrachloride
GO	gene ontology
KEGG	Kyoto Encyclopedia of Genes and Genomes
H&E	hematoxylin-eosin stain
qRT-PCR	quantitative reverse transcriptional polymerase chain reaction
C2cd2	C2 calcium-dependent domain containing 2
Ppm1f	protein phosphatase 1F
Slcla2	solute carrier family 1
Prom2	prominin 2
Prkaa2	protein kinase, AMP-activated, α 2 catalytic subunit
Marcksl1	MARCKS-like 1
Khk	ketohexokinase
Gabra3	γ-aminobutyric acid (GABA) A receptor, subunit α 3
Col9a3	collagen, type IX, α 3
TCA	tricarboxylic acid
Fbxl22	F-box and leucine-rich repeat protein 22
Hh	hedgehog
TNF	tumor necrosis factor
Bmp4	bone morphogenetic protein 4
Csnk1g1	casein kinase 1 γ 1
Gas1	growth arrest specific 1
Gli1	GLI-Krüppel family member 1
Hhip	Hh-interacting protein
Ihh	Indian hedgehog
cAMP	cyclic adenosine monophosphate
Prkacb	cAMP-dependent protein kinase catalytic subunit β
Stk36	serine/threonine kinase 36
Sufu	suppressor of fused homolog
MMTV	mouse mammary tumor virus
Wnt	wingless-type MMTV integration site family
MAPK	mitogen-activated protein kinase
mTOR	mechanistic target of rapamycin
HIF	hypoxia-inducible factor
FASN	fatty acid synthase
HMGCR	3-hydroxy-3-methylglutaryl-CoA reductase
MMP9	matrix metallopeptidase 9
DMN	dimethylnitrosamine
MCD	methionine/choline-deficient
NBF	neutral buffered formalin
RNU1A	U1A small nuclear RNA
SEM	standard error of the mean
GO	gene ontology
